# SARS-CoV-2 seroprevalence among parturient women in Philadelphia

**DOI:** 10.1126/sciimmunol.abd5709

**Published:** 2020-07-29

**Authors:** Dustin D. Flannery, Sigrid Gouma, Miren B. Dhudasia, Sagori Mukhopadhyay, Madeline R. Pfeifer, Emily C. Woodford, Jeffrey S. Gerber, Claudia P. Arevalo, Marcus J. Bolton, Madison E. Weirick, Eileen C. Goodwin, Elizabeth M. Anderson, Allison R. Greenplate, Justin Kim, Nicholas Han, Ajinkya Pattekar, Jeanette Dougherty, Oliva Kuthuru, Divij Mathew, Amy E. Baxter, Laura A. Vella, JoEllen Weaver, Anurag Verma, Rita Leite, Jeffrey S. Morris, Daniel J. Rader, Michal A. Elovitz, E. John Wherry, Karen M. Puopolo, Scott E. Hensley

**Affiliations:** 1 Division of Neonatology, Children’s Hospital of Philadelphia, Philadelphia, PA.; 2 Department of Pediatrics, University of Pennsylvania Perelman School of Medicine, Philadelphia, PA.; 3 Center for Pediatric Clinical Effectiveness, Children’s Hospital of Philadelphia, Philadelphia, PA.; 4 Department of Microbiology, University of Pennsylvania Perelman School of Medicine, Philadelphia, PA.; 5 Division of Infectious Diseases, Children’s Hospital of Philadelphia, Philadelphia, PA.; 6 Institute for Immunology, University of Pennsylvania Perelman School of Medicine, Philadelphia, PA.; 7 Department of Systems Pharmacology and Translational Therapeutics, University of Pennsylvania, Philadelphia, PA.; 8 Division of Gastroenterology, Department of Medicine, University of Pennsylvania Perelman School of Medicine, Philadelphia, PA.; 9 Institute for Translational Medicine and Therapeutics, University of Pennsylvania Perelman School of Medicine, Philadelphia, PA.; 10 Departments of Genetics and Medicine, Perelman School of Medicine, University of Pennsylvania, Philadelphia, PA.; 11 Maternal and Child Health Research Center, Department of Obstetrics and Gynecology, University of Pennsylvania Perelman School of Medicine, Philadelphia, PA.; 12 Department of Biostatistics Epidemiology and Informatics, University of Pennsylvania, Philadelphia, PA.

## Abstract

Limited data are available for pregnant women affected by SARS-CoV-2. Serological tests are critically important for determining SARS-CoV-2 exposures within both individuals and populations. We validated a SARS-CoV-2 spike receptor binding domain serological test using 834 pre-pandemic samples and 31 samples from COVID-19 recovered donors. We then completed SARS-CoV-2 serological testing of 1,293 parturient women at two centers in Philadelphia from April 4 to June 3, 2020. We found 80/1,293 (6.2%) of parturient women possessed IgG and/or IgM SARS-CoV-2-specific antibodies. We found race/ethnicity differences in seroprevalence rates, with higher rates in Black/non-Hispanic and Hispanic/Latino women. Of the 72 seropositive women who also received nasopharyngeal polymerase chain reaction testing during pregnancy, 46 (64%) were positive. Continued serologic surveillance among pregnant women may inform perinatal clinical practices and can potentially be used to estimate exposure to SARS-CoV-2 within the community.

## Teaser

Over six percent of parturient women in Philadelphia (4 April–3 June 2020) had serological evidence of exposure to SARS-CoV-2.

## INTRODUCTION

Severe acute respiratory syndrome coronavirus 2 (SARS-CoV-2) can cause serious disease in adult populations, particularly in those with underlying health conditions (*1*). Serological tests are important for determining SARS-CoV-2 viral exposures within individuals and populations (*2*). However, many commercial tests have high false positive rates and therefore cannot be used to accurately estimate seroprevalence in populations with relatively low levels of exposures (*3*, *4*). Serological tests are especially important for vulnerable populations such as pregnant women, because immune status has implications for management of both the pregnant woman and the newborn. Admission to the hospital for delivery is one of the few instances in which otherwise healthy individuals are consistently interacting with the medical system, and therefore provides an opportunity for surveillance of SARS-CoV-2 serology in the community.

We performed a study of pregnant women presenting for delivery from April 4 to June 3, 2020 at two academic birth hospitals in Philadelphia, Pennsylvania. Both hospitals are active clinical and research centers affiliated with the University of Pennsylvania, and combined represent 50% of live births in Philadelphia (*5*). Discarded maternal sera from delivery admission were collected, de-identified, and tested by enzyme-linked immunosorbent assay (ELISA) for SARS-CoV-2 immunoglobulin G (IgG) and immunoglobulin M (IgM) antibodies to the spike receptor binding domain (RBD) antigen.

## RESULTS

### Demographics

**Fig. 1:**
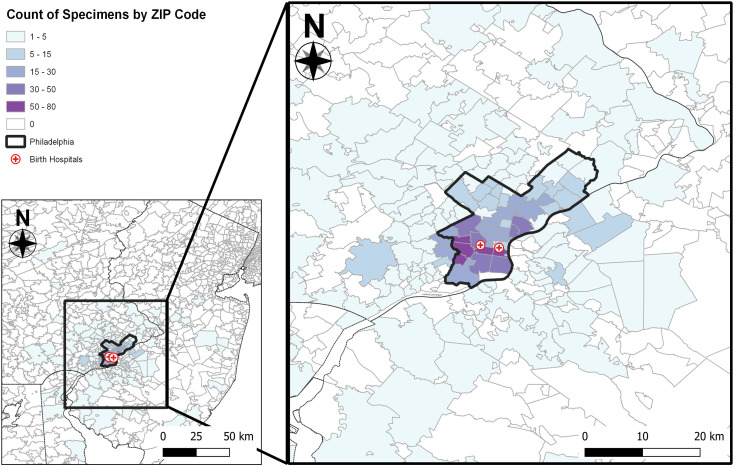
**Geographical distribution of women tested for SARS-CoV-2 antibodies**. Most serum specimens analyzed were from women living in areas within or immediately bordering the city of Philadelphia. Location of birth hospitals where serum samples were collected are shown as red crosses.

Demographics and clinical characteristics of the women are shown in Table 1. Most serum specimens were derived from women living in areas within or immediately bordering the city of Philadelphia (Fig. 1). Pregnant women who were symptomatic or exposed to SARS-CoV-2 underwent SARS-CoV-2 nasopharyngeal nucleic acid polymerase chain reaction (PCR) testing from April 4-12, 2020; universal PCR testing was recommended for all pregnant women presenting for delivery starting April 13, 2020. Of 1,620 women who delivered during the study period, 1,293 (80%) had available discarded serum specimens and were included in the analysis.

**Table 1:** Demographics and clinical characteristics of the study cohort.

**Characteristics**	**Total** **(n = 1,293)**	**Seropositive^1^** **(n = 80)**	**Seronegative** **(n = 1,213)**	**p-value^2^**
**Age (in years), median (IQR)**	31 (27, 35)	28 (24, 32)	31 (27, 35)	<0.001
**Race/ethnicity, n (%)^3^**				
**Black/Non-Hispanic**	537	52 (9.7)	485 (90.3)	<0.001
**White/Non-Hispanic**	447	9 (2.0)	438 (98.0)	<0.001
**Hispanic/Latino**	125	13 (10.4)	112 (89.6)	0.04
**Asian**	106	1 (0.9)	105 (99.1)	0.01
**Other/Unknown^4^**	78	5 (6.4)	73 (93.6)	0.93
**Pre-pregnancy BMI^5^, n (%)^3^**				
**Overweight (25.0 to <30.0)**	345	28 (8.1)	317 (91.9)	0.07
**Obese (≥30.0)**	337	27 (8.0)	310 (92.0)	0.09
**Diabetes^6^, n (%)^3^**	113	10 (8.9)	103 (91.1)	0.22
**Hypertension^6^, n (%)^3^**	404	33 (8.2)	371 (91.8)	0.05
**Asthma^6^, n (%)^3^**	194	13 (6.7)	181 (93.3)	0.75
**Cesarean delivery, n (%)^3^**	400	30 (7.5)	370 (92.5)	0.19
**Preterm delivery at gestational age <37 weeks, n (%)^3^**	128	11 (8.6)	117 (91.4)	0.23
**Live-born infant, n (%)^3^**	1,282	79 (6.2)	1,203 (93.8)	0.51

^1^Seropositivity was based on either IgG or IgM level >0.48 arbitrary units. ^2^Difference in maternal age was tested using Mann-Whitney U test, differences in proportion of all other characteristics were tested using χ^2^ tests or Fisher’s exact test as appropriate. For race/ethnicity and pre-pregnancy BMI, difference was tested at each level of the characteristic (e.g., proportion of Black women who were seropositive compared to proportion of non-Black women who were seropositive). ^3^Row percentages are shown which represent the percent of total in each characteristic (e.g., 9.7% of Black/Non-Hispanic women were seropositive). ^4^Race/ethnicity was unknown for 2 seropositive and 26 seronegative women; race was abstracted from documentation at time of admission and in clinical practice, is usually self-reported. ^5^Pre-pregnancy BMI was missing for 2 seropositive and 12 seronegative women; pre-pregnancy BMI was abstracted from documentation in the medical record, or from patient’s self-reported entry in birth registration. ^6^Diagnoses were based on delivery admission International Classification of Diseases, 10th revision diagnosis codes for diabetes (O24, E08-E13, Z79.4), hypertension (O10, O11, O13-O16, I10-I13, I15), and asthma (J45). BMI, body mass index; IQR, interquartile range.

### Assay validation

**Fig. 2:**
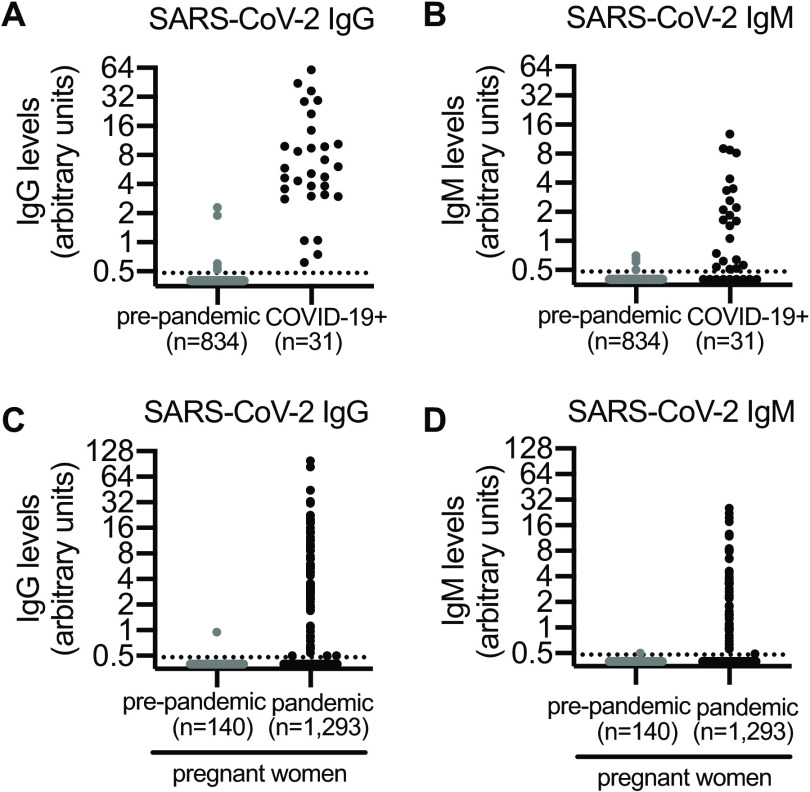
Serum SARS-CoV-2 antibody levels in COVID-19 pandemic and pre-pandemic individuals. (A-B) Relative levels of SARS-CoV-2 IgG (A) and IgM (A) in serum collected before the COVID-19 pandemic (n = 834) and serum collected from COVID-19 recovered donors (n = 31). (C-D) Relative levels of SARS-CoV-2 IgG (C) and IgM (D) in serum collected from pregnant women from 2009-2012 (n = 140) and from April 4-June 3, 2020 (n = 1,293). Dashed lines indicate 0.48 arbitrary units, which was used to distinguish positive versus negative samples (see Methods). Serum samples that were below the cutoff for seropositivity were assigned an antibody level of 0.40 arbitrary units.

Our serological assay utilized a SARS-CoV-2 spike RBD antigen and modified ELISA protocol first described by Amanat *et al*. (*6*). We validated this serological assay by testing serum samples collected prior to the pandemic in 2019 from 834 individuals in the Penn Medicine Biobank and 31 individuals who recovered from confirmed coronavirus disease 19 (COVID-19) infections in 2020 (Fig. 2A-B). All 31 serum samples from COVID-19 recovered donors contained high, but variable, levels of SARS-CoV-2 IgG (Fig. 2A) and 22 of 31 samples contained detectable levels of SARS-CoV-2 IgM (Fig. 2B). Conversely, only 5 of 834 samples collected before the pandemic contained SARS-CoV-2 IgG and only 4 of 834 samples contained SARS-CoV-2 IgM; none contained both IgG and IgM. Based on these data, the estimated sensitivity of the test is 100% (95% CI 89.1-100.0%) and the specificity is 98.9% (95% CI 98.0-99.5%). Using this test, we have found that there is heterogeneity in antibody responses among hospitalized COVID-19 patients and that some actively infected patients are seronegative (*7*, *8*). Consistent with our initial validation experiments, only 1 of 140 samples collected from pregnant women before the pandemic (from 2009-2012) possessed IgG or IgM SARS-CoV-2 antibodies (Fig. 2C-D).

### Serological findings

We found that 80 of 1,293 (seropositivity rate 6.2%, 95% CI [4.9-8.0%]) pregnant women presenting for delivery from April 4 to June 3, 2020 possessed IgG and/or IgM SARS-CoV-2 antibodies (Fig. 2C-D; p = 0.003 comparing samples from pre-pandemic and pandemic pregnant women). We identified 55 women with both SARS-CoV-2 IgG and IgM, 21 women with only SARS-CoV-2 IgG, and 4 women with only SARS-CoV-2 IgM (Table S1). SARS-CoV-2 antibody levels in samples from these women were variable (Fig. 2C-D), similar to what we found in samples from individuals recovering from confirmed SARS-CoV-2 infections (Fig. 2A-B). The seroprevalence rate was not statistically different comparing women living within the city limits of Philadelphia (62/986, 6.3%, 95% CI [4.9-8.0%]) to those living in surrounding areas in Pennsylvania (12/191, 6.3%, 95% CI [3.3-10.7%]), or surrounding areas in New Jersey (5/107, 4.7%, 95% CI [1.5-10.6%]). There were no significant differences in seroprevalence rates in women with or without comorbidities, preterm delivery, or Cesarean mode of delivery (Table 1). In contrast, we observed significant race/ethnicity differences in seroprevalence rates with higher rates in Black/non-Hispanic (9.7%, 95% CI [7.3-12.5%]) and Hispanic/Latino (10.4%, 95% CI [5.7-17.1%]) women and lower rates in White/non-Hispanic (2.0%, 95% CI [0.9-3.8%]) and Asian (0.9%, 95% CI [0.0-5.1%]) women (Table 1).

Nasopharyngeal swabs from 1,109 (85.8%) women were tested by SARS-CoV-2 PCR during the pregnancy or at the time of delivery. The majority of sera tested for antibody were obtained before or within 6 days of PCR testing (Table 2). SARS-CoV-2 antibodies were detected in all sera obtained from PCR-positive women when serum samples were obtained more than 7 days after PCR testing (Table 2). Overall, we found that 46 of 72 seropositive women who were tested by PCR had a SARS-CoV-2 positive PCR result, whereas only 18 of 1,037 seronegative women who were tested by PCR had a SARS-CoV-2 positive PCR result. While all serum samples were collected during the delivery admission, nasopharyngeal samples were collected at variable times either during the delivery admission or earlier in the pregnancy, and therefore, further study will be required to evaluate the temporal relationship between SARS-CoV-2 seropositivity and PCR positivity in pregnant women.

**Table 2:** Timing of serology testing and seropositivity with respect to nasopharyngeal PCR testing^1^.

**Serology Timing**	**NP-PCR Positive**		**NP-PCR Negative**	
	**Tested**	**Seropositive (%)**	**Tested**	**Seropositive (%)**
**Before NP-PCR test**	17	10 (58.8)	364	9 (2.5)
**0-6 days after NP-PCR test**	26	15 (57.7)	647	16 (2.5)
**7-13 days after NP-PCR test**	5	5 (100.0)	8	0
**14-20 days after NP-PCR test**	2	2 (100.0)	7	0
**≥21 days after NP-PCR test**	14	14 (100.0)	19	1 (5.3)
**Total**	64	46 (71.9)	1,045	26 (2.5)

^1^The table includes 1,109 women tested for serology who were also tested by nasopharyngeal PCR anytime during pregnancy up to discharge from delivery admission. NP, nasopharyngeal; PCR, polymerase chain reaction.

## DISCUSSION

Large-scale serology testing is critical for estimating how many individuals have been infected during the COVID-19 pandemic. Due to widely-imposed social distancing requirements, and to decreases in on-site, discretionary medical care, it is currently difficult to collect serum for population-wide serological testing. The vast majority of pregnant women, however, continue to have multiple interactions with the medical system for prenatal care and for delivery during this pandemic, and therefore present an opportunity to consistently assess SARS-CoV-2 exposures within a community. Our data suggest that 6.2% of parturient women in Philadelphia from April 4 to June 3, 2020 were previously exposed to SARS-CoV-2.

As of June 3, 2020, there were 23,160 confirmed cases of COVID-19 in the city of Philadelphia (*9*), which has a population size of nearly 1.6 million people. This suggests an infection rate of approximately 1.4%, which is more than 4 times lower than the estimates based on our serological data. Serologic studies may provide a more accurate means of assessing population exposure to SARS-CoV-2 by identifying asymptomatic or minimally symptomatic as well as symptomatic infections. Further studies are needed to determine how the immune status of pregnant women compares to that of the general population. For example, parturient women may not represent individuals of different ages within the general population and women and men might mount different antibody responses upon infection with SARS-CoV-2 (*10*).-Furthermore, most pregnant women cannot fully shelter-in-place during a pandemic as they continue to have interactions with the medical system. Our finding that Black/non-Hispanic and Hispanic/Latino women have higher SARS-CoV-2 seroprevalence rates relative to women of other races suggest that there are race/ethnicity differences in SARS-CoV-2 exposures in Philadelphia and surrounding areas. Identification of factors that contribute to such differences in exposure to SARS-CoV-2, including factors rooted in systemic racism, may inform public health measures aimed at preventing further infections (*11*–*13*).

Prior perinatal COVID-19 studies have primarily focused on virus detection (i.e., nucleic acid testing) in pregnant women and most of these studies have not evaluated antibody responses (*14*–*22*). Two published studies to date have assessed SARS-COV-2 serology in pregnant women with active disease. A study of 6 parturient women in Wuhan, China with confirmed COVID-19 found all 6 women had elevated levels of SARS-CoV-2 IgG and IgM (*23*). A case report from Peru detailed a symptomatic pregnant woman with positive PCR testing and negative serology at presentation, who developed severe respiratory failure necessitating delivery; her IgM and IgG turned positive 4 days after delivery (9 days after symptom onset) (*24*). Beyond describing individual response to infection, SARS-CoV-2 serological testing among pregnant women will be increasingly important for perinatal disease risk management, as well as for optimizing vaccine strategies when vaccines become available. Additional studies will be needed to address the impact of maternal infection on neonatal immune responses, and to determine those factors that may contribute to observed disparities in exposure to SARS-CoV-2.

## MATERIALS AND METHODS

### Study design

The goal of this study was to estimate SARS-CoV-2 seroprevalence rates in the community using discarded serum samples from parturient women. The Institutional Review Board at the University of Pennsylvania approved this study. There was a waiver of consent for testing of residual serum samples from parturient women, as indicated below. For other cohorts used to validate our assay, subjects were consented before samples were obtained. De-identified data were used for analysis.

### Serum samples from parturient women

Pregnant women at the two hospitals (Pennsylvania Hospital and Hospital of the University of Pennsylvania) have blood drawn for rapid plasma reagin (screening for syphilis per public health guidelines) testing as part of routine clinical care on admission to the hospital for delivery. Residual serum from this testing was obtained from the clinical laboratory at the time it was otherwise to be discarded. Demographic and clinical data were collected from review of electronic medical records to assess for differences in seroprevalence based on these factors. Race and ethnicity were self-reported. International Classification of Diseases, 10^th^ revision (ICD-10) diagnosis codes O24, E08-E13, Z79.4 were used to capture Type 1 diabetes, Type 2 diabetes and gestational diabetes; codes O10, O11, O13-O16, I10-I13, I15 were used to capture hypertensive disorders, gestational hypertension and pre-eclampsia; and code J45 was used to capture any history of asthma before or during pregnancy. To ensure these codes correctly captured patient condition, we manually reviewed records for the first 130 (10%) women with ICD-10 diagnosis of diabetes, hypertension or asthma; and reviewed a random sampling of 65 records from the first 130 (5% of total) women without any identified ICD-10 codes for these conditions. Patient numbers for the Table S1 were assigned at random. The Institutional Review Board at the University of Pennsylvania approved this study with waiver of consent.

### Serum samples from individuals recovered from COVID-19

Samples from subjects who had recovered from laboratory-confirmed SARS-CoV-2 infections were obtained at the University of Pennsylvania. Subjects were consented and samples obtained after laboratory-confirmed COVID-19 diagnosis and >14 days since resolution of symptoms. The Institutional Review Board at the University of Pennsylvania approved this study.

### Pre-pandemic human serum samples

To validate our serological assay, serum samples from 834 adults (19-89 years old; 52% females) were collected via the Penn Medicine Biobank (PMBB) between October and December of 2019, prior to the COVID-19 pandemic. PMBB routinely consents individuals visiting the University of Pennsylvania healthcare system and obtains and stores biospecimens. Banked serum samples obtained from pregnant women from 2009-2012 were also utilized as pre-pandemic controls. For these banked samples, maternal serum was collected during the third trimester of pregnancy as part of an IRB-approved study. From this study, 140 samples were randomly selected from women who delivered at term (average gestational age at time of sample collection was 33.8 weeks, 80% were Black women).

### Enzyme-linked immunosorbent assay (ELISA)

ELISAs were completed using plates coated with the receptor binding domain (RBD) of the SARS-CoV-2 spike protein using a previously described protocol with slight modifications. (*6*, *25*) Plasmids for expressing this protein were provided by Florian Krammer (Mt. Sinai). SARS-CoV-2 RBD proteins were produced in 293F cells and purified using nickel-nitrilotriacetic acid (Ni-NTA) resin (Qiagen). The supernatant was incubated for 2 hours with Ni-NTA resin at room temperature before the Ni-NTA resin was collected using gravity flow columns and the protein was eluted. After buffer exchange into phosphate-buffered saline (PBS), the purified protein was stored in aliquots at -80°C. ELISA plates (Immulon 4 HBX, Thermo Scientific) were coated overnight at 4°C with 50 μL per well of PBS or a 2 μg/mL recombinant protein diluted in PBS. The next day, ELISA plates were washed 3 times with PBS containing 0.1% Tween-20 (PBS-T) and blocked for 1 hour with PBS-T supplemented with 3% non-fat milk powder. Prior to testing in ELISA, serum samples were heat-inactivated at 56°C for 1 hour. Serum samples were serially diluted in 2-fold in 96-well round-bottom plates in PBS-T supplemented with 1% non-fat milk powder (dilution buffer), starting at a 1:50 dilution. Next, ELISA plates were washed 3 times with PBS-T and 50 μL serum dilution was added to each well. Plates were incubated for 2 hours at room temperature using a plate mixer. Plates were washed again 3 times with PBS-T before 50 μL of horseradish peroxidase (HRP) labeled goat anti-human IgG (Jackson ImmunoResearch Laboratories) (1:5,000) or goat anti-human IgM-HRP (SouthernBiotech) (1:1,000) secondary antibodies were added. After 1 hour incubation at room temperature using a plate mixer, plates were washed 3 times with PBS-T and 50 μL SureBlue 3,3′,5,5′-tetramethylbenzidine (TMB) substrate (KPL) was added to each well. Five minutes later, 25 μL of 250 mM hydrochloric acid was added to each well to stop the reaction. Plates were read at an optical density (OD) of 450 nm using the SpectraMax 190 microplate reader (Molecular Devices). Background OD values from the plates coated with PBS were subtracted from the OD values from plates coated with recombinant protein. A dilution series of the IgG monoclonal antibody CR3022, which is reactive to the SARS-CoV-2 spike protein, was included on each plate as a control to adjust for inter assay variability. The IgG CR3022 monoclonal antibody was included on both IgG and IgM plates, and an anti-human IgG-HRP secondary antibody was added to these standardization wells on both IgG and IgM plates. In essence, the CR3022 monoclonal antibody was used to set the OD threshold on each plate and to ensure that the same OD threshold was used on all plates, including both IgG and IgM assays. Serum antibody concentrations were reported as arbitrary units relative to the CR3022 monoclonal antibody. Plasmids to express the CR3022 monoclonal antibody were provided by Ian Wilson (Scripps). All samples were first tested in duplicate at a 1:50 serum dilution. Samples with an IgG and/or IgM concentration above the lower limit of detection (0.20 arbitrary units) were repeated in at least a 7-point dilution series to obtain quantitative results.

### Establishment of an ELISA cutoff to distinguish seropositive versus seronegative

We used results from the 2019 cohort (Fig. 2A) to set ELISA cutoffs for seropositivity and seronegativity. Over the course of establishing our serological assay, we identified rare individuals who possessed pre-pandemic SARS-CoV-2 cross-reactive serum antibodies. Most of these individuals possessed very low levels of cross-reactive SARS-CoV-2 antibodies. We found that ~1% of samples from the pre-pandemic 2019 cohort had IgG and/or IgM levels of >0.48 arbitrary units, which was subsequently used as the cutoff for defining seropositivity in the 2020 cohort.

### Statistical methods

Standard descriptive analyses using χ^2^ test, Fisher’s exact test, and Mann-Whitney U test as appropriate, compared the demographic and clinical characteristics between the seropositive and seronegative women. Confidence intervals for proportions were computed using the Clopper-Pearson (exact) method. Statistical significance was set at p-value <0.05. Statistical analyses were performed using Stata version 16 (StataCorp, College Station, TX) and Prism version 8 (GraphPad Software). Figure 1 was created using QGIS version 3.12.3.

## Supplementary Materials

immunology.sciencemag.org/cgi/content/full/5/49/eabd5709/DC1 Table S1. Relative levels of SARS-COV-2 IgG and IgM in serum collected from seropositive pregnant women (n = 80).
